# First Report of Porcine Bocavirus and Porcine Cytomegalovirus in Croatian ASF-Negative Wild Boar Populations

**DOI:** 10.3390/v18070693

**Published:** 2026-06-23

**Authors:** Jelena Prpić, Magda Kamber Taslaman, Margarita Božiković, Daria Jurković Žilić, Andreja Jungić, Ivana Lojkić, Lorena Jemeršić

**Affiliations:** 1 Department of Virology, Croatian Veterinary Institute, 10000 Zagreb, Croatia; kamber@veinst.hr (M.K.T.); jungic@veinst.hr (A.J.); ilojkic@veinst.hr (I.L.); jemersic@veinst.hr (L.J.); 2 Department of Bacteriology and Parasitology, Croatian Veterinary Institute, 10000 Zagreb, Croatia; jurkovic@veinst.hr

**Keywords:** wild boar, porcine bocavirus, porcine cytomegalovirus, wildlife surveillance, Croatia, viral circulation, coinfection

## Abstract

Wild boar populations are increasingly recognized as important hosts in the ecology of swine viruses, yet data from Croatia remain limited. This study aimed to establish baseline information on the presence of porcine bocavirus (PBoV) and porcine cytomegalovirus (PCMV) in Croatian wild boar within the framework of the national African swine fever (ASF) surveillance program. Spleen and blood samples from 184 ASF-negative wild boar collected across 11 counties were tested using real-time PCR. PCMV DNA was detected in 16 animals (8.69%), with similar detection frequencies in spleen (7.69%) and blood (9.52%). PBoV DNA was identified in seven animals (3.80%), all from spleen samples. Positive animals were distributed across several counties, but no significant associations were observed between virus detection and age, sex, or geographic origin. Coinfection with both viruses was detected in a single animal (0.05%). These findings provide the first molecular evidence of PBoV and PCMV in Croatian wild boar and indicate low-level viral circulation across multiple regions. Although both viruses are typically subclinical, their detection contributes to understanding pathogen diversity in free-living suids and establishes a foundation for future epidemiological and molecular studies in the region.

## 1. Introduction

Wild boar (*Sus scrofa*) populations have expanded significantly across Europe in recent decades due to changes in land use, reduced predation, climate change, and increased food availability [1,2]. As their numbers rise, so does their role as reservoirs and amplifying hosts for numerous viral, bacterial, and parasitic pathogens relevant to both wildlife and domestic animal health [3]. Their frequent use of agricultural landscapes, high mobility, and dense local populations make wild boar a key species in the epidemiology of several swine diseases [4]. Consequently, many European countries, including Croatia, have incorporated wild boar into national surveillance programs, particularly those focused on African swine fever (ASF) [5]. These programs also offer opportunities to investigate additional pathogens circulating in wild boar populations [6].

Among the viruses increasingly detected in wild boar are porcine bocavirus (PBoV) and porcine cytomegalovirus (PCMV), both of which are considered relevant from a wildlife health perspective because they may influence subclinical infection dynamics and contribute to broader pathogen circulation [7,8]. However, their true prevalence in free-living populations remains poorly characterized [9,10], with only a limited number of regional studies available. Studying their interrelation provides a more complete picture of viral diversity at the wildlife–livestock interface. For example, PBoV was identified in 12.94% of Romanian wild boar [9], while PCMV has been reported in countries such as Italy and Germany [10], though the published data remain insufficient for broader prevalence estimates.

PBoV, a member of the family *Parvoviridae*, was first described in domestic pigs in 2009 [11] and is now recognized globally. Although often subclinical, it has a broad range of tissue tropism [12] and is associated with respiratory, reproductive, and enteric disorders that may cause high mortality in newborn piglets and those up to three weeks of age [13]. Co-infections with other viruses and microbes may worsen clinical outcomes [14]. High detection rates in domestic pigs and wild boar across Europe suggest that PBoV is well established in the family *Suide* [8]. Its frequent co-circulation with other pathogens, including porcine circovirus (PCV) and porcine reproductive and respiratory virus (PRRSV), has raised questions about its contribution to multifactorial disease complexes [15].

PCMV, a betaherpesvirus within the family *Herpesviridae* [16], is widespread in domestic pigs and wild boar. Although infections are typically latent or mild [17], PCMV has gained prominence due to its impact on xenotransplantation, where transmission from donor pigs to non-human primates has been associated with reduced graft survival and heightened inflammatory responses [18,19]. In addition to its relevance in xenotransplantation, PCMV is known to participate in co-infections and can modulate host immune responses, potentially facilitating secondary viral or bacterial infections, which further underscores its importance in free-living and domestic pig populations. In Europe, PCMV has been documented in wild boar populations in several countries, where it may circulate independently or alongside domestic pig populations [10,20].

Currently, despite these findings, information on PBoV and PCMV in Croatia is lacking. No molecular data have been available for both viruses in the Croatian wild boar, and no serological surveys have been conducted. The selection of PBoV and PCMV for this study was determined by the type of material available through the national ASF surveillance program, which provides only blood and spleen samples collected postmortem. These matrices are not suitable for reliable detection of PRRSV or influenza A virus in wild boar, and expanding the pathogen panel was therefore not feasible within the constraints of the existing surveillance framework. Given that no molecular data existed for either PBoV or PCMV in Croatia, the primary aim of this study was to establish baseline information for these two viruses using the material that was systematically available. Some insight, however, comes from domestic pigs. It was demonstrated that PBoV is already widespread in Croatian swine herds [21], with viral DNA detected in 42.1% of pooled samples from commercial farms and 27.9% from backyard pigs. Sequencing revealed multiple lineages closely related to strains circulating in Europe, Asia, and North America. In neighboring countries such as Slovenia, Hungary, and Bosnia and Herzegovina, limited or fragmented data have been documented [22], further constraining regional epidemiological understanding. PCMV has recently been detected at high prevalence in wild boar in Serbia, where 74.2% of tested animals were positive [20], indicating substantial circulation in the Western Balkans. Given the absence of natural barriers to wild boar movement, a similar viral presence in Croatia is possible. This study was designed as a baseline molecular survey, and genomic sequencing was beyond the scope of the available material and funding. Although phylogenetic analysis would provide valuable insight into the relationship between Croatian strains and those circulating in neighboring countries, the ASF surveillance framework provides limited sample types and does not include resources for downstream sequencing. Future targeted sampling efforts, supported by dedicated resources, will enable genetic characterization and more detailed epidemiological interpretation.

Establishing baseline data on these viruses in Croatian wild boar is therefore essential for assessing transmission risks, understanding epidemiological patterns, and supporting future wildlife health monitoring. Within the framework of the national ASF surveillance program, we analyzed spleen and blood samples from wild boar across several Croatian counties using real-time PCR to detect PBoV and PCMV. Since ASF surveillance requires systematic sampling of wild boar carcasses, it provides an efficient platform for opportunistic testing of additional pathogens. To our knowledge, this study provides the first molecular evidence of both viruses in Croatian wild boar. By documenting their detection and describing their demographic and geographic distribution, this work contributes new insight into the viral landscape of wild boar in Croatia and establishes a foundation for future epidemiological investigations. Because both viruses are widespread, often subclinical, and capable of co-circulating with other swine pathogens, assessing their presence in wild boar contributes to understanding broader pathogen dynamics in free-living swine populations. The objective of this study was to determine the presence and distribution of PBoV and PCMV in Croatian wild boar using molecular methods and to generate baseline data for subsequent assessments.

## 2. Materials and Methods

### 2.1. Study Design and Sampling

**Figure 1:**
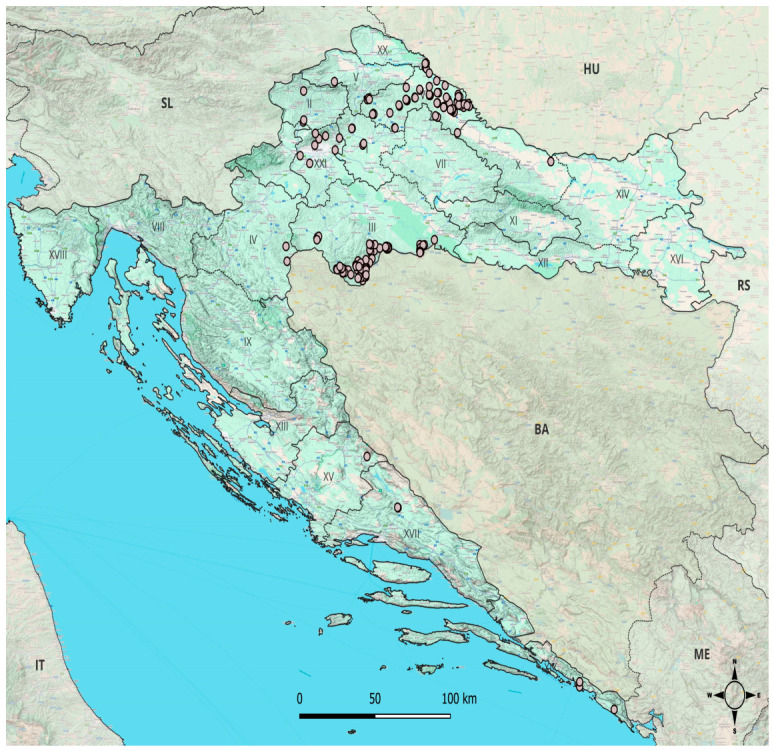
A map of Croatia that displays the distribution of wild boar sampling sites by county. Roman numerals (I–XXI) are used to represent counties on the map as follows: I. Zagreb County; II. Krapina–Zagorje; III. Sisak–Moslavina; IV. Karlovac; V. Varaždin; VI. Koprivnica–Križevci; VII. Bjelovar–Bilogora; VIII. Primorje–Gorski Kotar; IX. Lika–Senj; X. Virovitica–Podravina; XI. Požega–Slavonia; XII. Brod–Posavina; XIII. Zadar; XIV Osijek-Baranja; XV. Šibenik–Knin; and XVI. Split–Dalmatia, XVIII; Istria, XIX; Dubrovnik–Neretva, XX; Vukovar–Srijem, XVII. Međimurje, XXI. Zagreb County City. Bosnia and Herzegovina (BA), Hungary (HU), Montenegro (ME), Serbia (RS), and Slovenia (SI) are Croatia’s international neighbors; Italy (IT) and Croatia share a maritime boundary in the Adriatic Sea. Sampled samples were mapped using QGIS software version 3.30.0.

The spleen and blood samples were collected from wild boar hunted during the 2026 hunting season between January and April, according to an ongoing national African swine fever surveillance program prescribed by the Veterinary and Food Safety Directorate, the Croatian Ministry of Agriculture, Forestry, and Fisheries. All sampling activities complied with Croatian hunting legislation and the relevant ASF control framework, including the Regulation on Control Measures for ASF in Croatia (OG 124/25), Regulation (EU) 2016/429, Delegated Regulation (EU) 2020/687, Implementing Regulation (EU) 2023/594, and the National Animal Health Act (OG 152/22, 154/22). Only samples collected through the national ASF surveillance program that contained sufficient spleen tissue and/or whole blood for molecular analysis were included in the study. Carcasses in advanced decomposition, samples with inadequate tissue quantity or quality, and samples with missing or incomplete metadata (age, sex, or location) were excluded from the analysis to ensure consistency and reliability of the dataset. Samples were collected from 11 counties (Table 1): Koprivnica–Križevci, Zagreb, Varaždin, Krapina–Zagorje, Šibenik–Knin, Split–Dalmacija, Dubrovnik–Neretva, Karlovac, Sisak–Moslavina, Virovitica–Podravina, and the City of Zagreb (Figure 1). All animals were sampled postmortem immediately after harvesting. Five milliliters of whole blood were collected from each wild boar carcass and transferred into sterile collection tubes without anticoagulant (DeltaLab, Rubi, Barcelona, Spain). Whole-blood samples were collected postmortem by puncturing the jugular vein immediately after carcass inspection. A spleen fragment of approximately 3 × 3 cm was excised from each animal and placed into 120 mL polypropylene screw-cap containers (DeltaLab, Rubi, Barcelona, Spain). Tissue and blood specimens were stored under chilled conditions and transported to the laboratory within 24 h for further processing.

A total of 80 blood and 104 spleen samples were collected. Whole-blood and spleen samples were not obtained from the same individuals; sample type availability depended on carcass condition and the sampling procedures applied within the national ASF surveillance program. Samples were classified into age categories of up to six months, six months to one year, one to two years, and over two years old. Age was estimated based on dentition characteristics [23]. The dataset included both male and female wild boar (Table 1).

### 2.2. Molecular Detection of DNA

For each animal, a single DNA extract was used for all molecular assays, including ASFV, PCMV, and PBoV real-time PCR. DNA was isolated from prepared serum and from spleen homogenates, depending on the sample type available for each animal. Serum was separated by centrifugation at 220× *g* for 10 min using a Rotina 420 centrifuge equipped with a four-place swing-out rotor (effective radius 16 cm; Hettich, Tuttlingen, Germany). The resulting serum fraction was stored at 4–8 °C until analysis. A spleen segment was homogenized immediately upon arrival at the laboratory in phosphate-buffered saline (PBS; pH 7.2) at a 1:10 ratio. The homogenate was vortexed for one minute and centrifuged at 220× *g* for five minutes. Supernatants were collected and stored at −20 °C until testing.

DNA was isolated using an automated protocol on the KingFisher Flex instrument (Thermo Fisher Scientific, Waltham, MA, USA) with the IndiMag Pathogen Kit (Indical Bioscience GmbH, Leipzig, Germany), following the manufacturer’s instructions, using prepared serum and spleen homogenate supernatants as input material. An exogenous non-target positive control (NTPC-ASF) was added to each reaction to verify the absence of PCR inhibition. A negative wild boar serum sample was processed alongside the study samples during extraction to monitor potential cross-contamination. The negative wild boar serum used as an extraction control originated from a previously tested wild boar sample that had consistently tested negative for ASFV, PCMV, and PBoV in routine diagnostics. In addition, a weak positive reference serum (RSPS), prepared as part of the assay validation, was included as a procedural control. DNA extracts that were not immediately analyzed by real-time PCR were stored at −20 °C until testing.

Real-time PCR was carried out on a CFX96 detection system (Bio-Rad, Hercules, CA, USA) using the ID Gene™ African Swine Fever Triplex Kit (IDvet, Grabels, France), following the manufacturer’s instructions. Each reaction was prepared in a total volume of 20 µL, which included 3 µL of extracted nucleic acid. An internal positive control (IPC), a positive amplification control (PAC-ASF), the reference weak positive serum (RSPS), and a no-template negative control were included in every run. The NTPC-ASF was supplied as part of the commercial kit and was used according to the manufacturer’s instructions. In contrast, the RSPS was prepared and routinely used in-house as part of our laboratory’s internal quality control system. Its performance is regularly monitored through participation in national ASF proficiency testing schemes, ensuring consistent and reliable assay validation. Samples with a cycle threshold (Ct) value below 35 were considered positive, in accordance with the validation criteria established by the kit manufacturer. Samples yielding Ct values ≥ 35 were interpreted as negative, and no retesting strategy was applied, as repeat testing is not recommended by the manufacturer for results above this threshold.

After ASF screening, samples were tested for porcine bocavirus (PBoV) and porcine cytomegalovirus (PCMV) using previously published primers [14,18], listed in Table 2. Reactions were prepared with the ORA™ qPCR Probe Mix 2× (HighQu GmbH, Kraichtal, Germany) in a final volume of 20 µL, containing 3 µL of DNA template, 500 nM each of forward and reverse primer, and 250 nM of probe. Slovenian PBoV-positive samples, kindly provided by Dr. Ivan Toplak (Veterinary Faculty, Ljubljana, Slovenia), and Serbian PCMV-positive samples, provided by Dr. Vesna Miličević (Scientific Institute of Veterinary Medicine of Serbia, Belgrade, Serbia), served as positive controls. Ultra-pure water was used as the negative control.

Thermal cycling conditions consisted of an initial activation step at 95 °C for 2 min, followed by 40 cycles of denaturation at 95 °C for 5 s and annealing/extension at 60 °C for 30 s. Samples with Ct values ≤ 38 were considered positive. This Ct threshold was adopted from previously published protocols for PBoV and PCMV detection, which define Ct ≤ 38 as the recommended positivity criterion. Samples with Ct values close to this threshold were not retested, as replicate confirmation is not required in these validated assays.

### 2.3. Statistical Analysis

Statistical analyses were performed to describe the occurrence of PBoV and PCMV in ASF-negative wild boar and to explore potential associations with demographic and geographic factors. Virus prevalence was calculated as the proportion of PCR-positive samples among all tested animals, and 95% confidence intervals were estimated using the Wilson method. Differences in detection frequencies between age categories, sex, and sampling counties were evaluated using the chi-square test. The assumptions of the chi-square test were verified prior to analysis, and the test was applied only when all expected cell counts met the required threshold. When expected frequencies were <5, Fisher’s exact test was used instead. The relationship between PBoV and PCMV detection was assessed by comparing the distribution of single-positive and co-positive animals using Fisher’s exact test. Statistical significance was defined as *p* < 0.05. Statistical analyses were performed using standard online calculators [24], which implement Wilson confidence intervals, chi-square tests, and Fisher’s exact tests based on established statistical methods.

## 3. Results

### 3.1. Molecular Detection of ASFV, PCMV and PBoV in Wild Boar Samples

A total of 184 wild boars were included in the study. Of these, 97 were males (52.72%), and 87 were females (47.28%). Animals were assigned to five age categories: (1) up to 6 months (n = 18), (2) 6 months to 1 year (n = 51), (3) 1 to 2 years (n = 51), (4) older than 2 years (n = 46), and (5) age not determined because hunters did not report age (n = 18). The largest proportion of sampled animals belonged to age groups 2 and 3, each representing 27.72% of the total sample.

To exclude biased results, all samples tested negative for ASFV using the ID Gene^®^ ASF Triplex real-time PCR assay. Amplification curves were consistently observed in the HEX and Cy5 channels for every sample, confirming proper performance of both the extraction control and the internal amplification control. These signals indicate that neither nucleic acid isolation nor the qPCR reaction was inhibited and that the negative results obtained for the ASFV targets can be interpreted with confidence. The same DNA extracts were subsequently used for the PBoV and PCMV assays; therefore, the absence of inhibition demonstrated in the ASFV assay supports the reliability of the negative results obtained for these two targets, despite the lack of assay-specific internal controls.

PCMV DNA was identified in 8 of 104 spleen samples (7.69%) and in 8 of 84 blood samples (9.52%), resulting in an overall prevalence of 8.69% (16/184). The spatial distribution of PCMV-positive animals, together with their sex, age category, and sample type, is summarized in Table 3.

PBoV DNA was detected in 7 of 104 spleen samples (6.73%), corresponding to an overall prevalence of 3.80% (7/184). Details on the distribution of PBoV-positive animals across counties and hunting grounds, including demographic data and sample type, are presented in Table 4.

Coinfection with PCMV and PBoV was detected in only one blood sample, collected from a male wild boar of undetermined age from Koprivnica–Križevci County (hunting ground VI/107, Prigorje). This corresponds to a coinfection prevalence of 0.05% (1/184).

**Figure 2:**
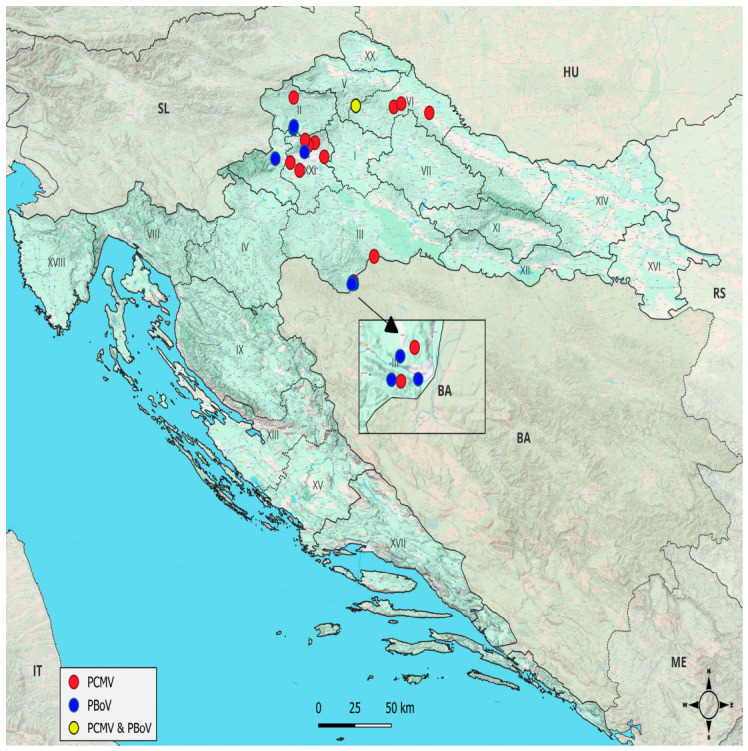
Map of Croatia displaying the hunting grounds where PCMV- and/or PBoV-positive wild boar were identified. Positive samples were mapped using QGIS software version 3.30.0.

The spatial distribution of PCMV-positive or PBoV-positive wild boars is shown in Figure 2.

### 3.2. Statistical Results

Analysis of the 184 ASF-negative wild boars revealed low but measurable circulation of both viruses in the sampled population. PCMV was detected in 16 animals (8.69%; 95% CI: 5.4–13.7%), while PBoV was identified in 7 animals (3.80%; 95% CI: 1.8–7.6%). When stratified by sample type, PCMV was found in 8 of 104 spleen samples (7.69%; 95% CI: 4.0–14.4%) and in 8 of 84 blood samples (9.52%; 95% CI: 4.9–17.6%). The difference in detection frequency between blood and spleen was not statistically significant (*p* > 0.05).

No significant associations were observed between PCMV detection and sex or age category (*p* > 0.05). The distribution of positives across counties also did not differ significantly from expectation, reflecting the small number of detections per location. For PBoV, statistical comparisons were limited by the low number of positive animals (n = 7). Descriptive evaluation showed that detections occurred in two counties, but no meaningful statistical differences across demographic groups could be assessed.

Geographic analysis showed that PCMV occurred in four counties, whereas PBoV was restricted to two. Despite these apparent differences in spatial spread, statistical testing did not identify significant geographic clustering for either virus. The limited number of positive detections reduced the power to detect subtle spatial trends, but the available data suggest that both viruses are present at low levels across multiple regions rather than being confined to specific hotspots.

Coinfection analysis showed that only one animal tested positive for both viruses. Fisher’s exact test indicated no significant association between PBoV and PCMV detection (*p* > 0.05), consistent with independent circulation patterns. Overall, the statistical evaluation supports the descriptive nature of the findings: both viruses were detected at low prevalence, and no statistically significant demographic or geographic trends were identified within the available dataset.

## 4. Discussion

This study provides the first molecular confirmation of PCMV and PBoV in Croatian wild boar, establishing an initial baseline for understanding their circulation in free-living swine populations. The study relied on blood and spleen samples because these are the only tissues consistently collected within the ASF surveillance system. Although both viruses exhibit broader tissue tropism, spleen and blood are commonly used matrices in wildlife studies and have been successfully applied in previous European surveys. Nevertheless, the prevalence values reported here should be interpreted as minimum estimates, as the use of additional tissues such as lymph nodes, tonsils, or respiratory and intestinal samples would likely increase detection sensitivity. Although both viruses were detected at low prevalence, their presence is epidemiologically relevant, given the increasing importance of wild boar in the maintenance and spread of swine pathogens across Europe. Contrary to previously published data [10,25], our findings show no statistical significance regarding the geographical distribution of the viruses, sex, or age of animals.

Understanding virus circulation in wild boar is essential for interpreting disease dynamics at the wildlife–livestock interface, particularly in regions where free-living and domestic populations overlap [7,26]. In Croatia, the wild boar is a well-known sentinel species for different viral diseases such as African swine fever [27] or, in the One Health (OH) context, hepatitis E [28,29]. Even so, molecular data from Croatia regarding PCMV and PBoV have been limited, making it difficult to position these infections nationally and within the broader European context [30]. To address this gap, the present study examined the occurrence of PBoV and PCMV in 11 different Croatian counties. By integrating these results with regional knowledge, this study assists in clarifying the role of Croatian wild boar in the wider ecology of swine viruses [4].

PCMV prevalence in this study was markedly lower than values reported in neighboring Serbia (74.2%) [20] and below those documented in Italy (36%) and Germany (60%) [10]. Seasonal timing may partly explain this discrepancy, as sampling was conducted during winter, whereas PCMV viral activity and shedding reportedly increase during late summer and autumn [31]. Even so, the detection of PCMV nonetheless adds an important component to the regional herpesvirus landscape. The pseudorabies virus (PRV), another herpesvirus of swine, has previously been detected in Croatia [32] and neighboring countries, such as Italy, Hungary, and Serbia [25,33], proving the permeability of wildlife populations across borders. Including PCMV in surveillance panels therefore reflects a more realistic picture of herpesvirus ecology in wild boar.

PBoV was detected at a lower prevalence (3.80%) than reported in the Romanian wild boar (12.94%) [9] and substantially below levels typically observed in domestic pigs across Europe (20–60%) [11,34]. This supports the view that domestic pigs act as the primary reservoir for PBoV, while wild boars likely play a secondary role in environmental maintenance [35]. In Croatia, PBoV is well documented in domestic pigs, with genetic diversity patterns suggesting repeated introductions and ongoing circulation [21]. The lower prevalence in wild boar populations is consistent with broader European trends [9,34].

No significant associations were found between virus detection and age, sex, or geographic origin. Although PCMV-positive animals were most frequently detected in the 1–2-year age group, this pattern did not differ from expectations and aligns with the biology of betaherpesviruses, which typically establish early-life infection followed by lifelong latency [10,31]. PBoV detections occurred across all age categories, consistent with its ability to infect pigs throughout life and often remain subclinical [14,34]. Because only seven animals tested positive for PBoV, the statistical power for detecting associations with demographic or geographic variables was limited. These findings should therefore be interpreted as descriptive rather than conclusive, and additional data will be required to assess potential epidemiological patterns.

Only one animal was coinfected with both PCMV and PBoV. Given the low prevalence of each virus, the probability of detecting coinfection was inherently low. The absence of such patterns here likely reflects the low viral burden in the studied population.

Although PCMV and PBoV are typically subclinical, their detection in wild boar remains relevant for both animal health and public health. Wild boars act as reservoirs and bridge hosts, facilitating pathogen exchange at the wildlife–livestock interface [26]. Monitoring PCMV is particularly important due to its implications for xenotransplantation, where latent infection in donor animals can compromise graft safety [36]. PBoV, meanwhile, is frequently identified in mixed infections and may contribute to multifactorial disease processes, especially when combined with immunomodulating viruses such as PCV2 [37,38]. Similar findings were observed in human bocavirus (HBoV) infections, indicating that over 50% of HBoV respiratory infections in children involve a co-infection with other viruses [39]. Therefore, integrating this work into the national ASF surveillance program demonstrates the practical value of multi-pathogen monitoring and highlights how existing infrastructure can be leveraged to obtain a more comprehensive picture of viral circulation in wild boar.

The epidemiological relevance of wild boars as reservoirs and amplifiers of viruses can be elaborated through zoonotic viral infections as well, such as influenza viruses or hepatitis E virus (HEV), which have been extensively studied in Croatia [28,29,40]. This parallel supports a broader principle: once a swine-associated virus becomes established in wild boar, it can form a sustainable sylvatic cycle that complicates control efforts and necessitates coordinated surveillance across sectors.

This is particularly relevant for Croatia, where wild boar populations move freely across the Dinaric–Pannonian region, a continuous ecological landscape without natural barriers to swine dispersal [26,41,42]. High PCMV circulation in Serbia increases the likelihood of transboundary viral movement through natural migration and overlapping habitats that allow environmental contamination and virus transmission. It is therefore possible that PCMV is more widespread in Croatian wild boar than currently detected, reflecting the absence of targeted surveillance rather than true rarity.

Several limitations regarding our results should be highlighted. The low number of positive detections reduces statistical power and limits the ability to detect subtle epidemiological patterns. Uneven sampling across counties may obscure spatial variation, and restricting diagnostics to spleen and blood likely underestimates true prevalence, particularly for viruses with broader tissue tropism or intermittent shedding. Future studies would benefit from larger and more evenly distributed sample sets, inclusion of additional tissues such as lymph nodes, tonsils or respiratory and gastrointestinal samples, and genetic characterization of detected strains. Comparative analyses involving domestic pigs and a more detailed investigation of coinfections would further clarify the role of wild boar in the maintenance and spread of swine viruses in this part of Europe.

## 5. Conclusions

This study documents the first molecular detection of PBoV and PCMV in Croatian wild boar and provides an initial reference point for assessing the circulation of these viruses in free-living swine populations. Although prevalence was low, the presence of both pathogens confirms that wild boar in Croatia participate in the broader regional ecology of swine viruses and should be considered within national surveillance and disease-prevention frameworks.

Given the high connectivity of wild boar populations across the Dinaric–Pannonian region, continued monitoring is warranted, especially in view of the significantly higher PCMV prevalence reported in neighboring countries. Expanded sampling, inclusion of additional tissues and age classes, and molecular characterization of detected strains will be essential for determining whether the low prevalence observed here reflects true epidemiological conditions or current surveillance limitations.

Future work should also examine interactions with other immunomodulating pathogens, such as PCV2 and PRRSV, to better understand the potential for synergistic effects and their implications for both wildlife and domestic pigs. Strengthening integrated wildlife–livestock surveillance will support more informed risk assessment and contribute to a clearer understanding of the role of wild boar in the maintenance and dissemination of swine viruses in this part of Europe.

## Figures and Tables

**Table 1:** Distribution of tested wild boar samples by county, gender, age class, and sample type.

County	Gender	Age Group	Sample Type	Number of Tested Animals
Koprivnica–Križevci	Male	<6 months	Blood	3
	Male	6 months–1 year	Blood	11
	Male	1–2 years	Blood	15
	Male	>2 years	Blood	5
	Male	Not determined	Blood	7
	Female	<6 months	Blood	5
	Female	6 months–1 year	Blood	10
	Female	1–2 years	Blood	10
	Female	>2 years	Blood	5
	Female	Not determined	Blood	1
	Female	Not determined	Spleen	4
Grad Zagreb	Male	6 months–1 year	Blood	1
	Male	1–2 years	Spleen	1
	Male	Not determined	Spleen	1
	Female	<6 months	Blood	1
	Female	6 months–1 year	Spleen	2
	Female	>2 years	Spleen	1
	Female	n.d.	Spleen	1
Zagreb	Male	Not determined	Spleen	1
	Female	>2 years	Spleen	1
Varaždin	Female	Not determined	Spleen	1
Krapina–Zagorje	Male	<6 months	Spleen	1
	Female	6 months–1 year	Spleen	2
Šibenik–Knin	Male	>2 years	Spleen	1
Split–Dalmacija	Male	<6 months	Spleen	1
	Male	6 months–1 year	Spleen	1
Dubrovnik–Neretva	Male	1–2 years	Blood	1
	Male	>2 years	Blood	1
	Female	>2 years	Blood	1
Karlovac	Male	6 months–1 year	Spleen	2
	Female	>2 years	Spleen	1
Virovitica–Podravina	Male	<6 months	Spleen	1
Sisak–Moslavina	Male	<6 months	Spleen	5
	Male	6 months–1 year	Spleen	8
	Male	1–2 years	Spleen	14
	Male	>2 years	Spleen	14
	Male	6 months–1 year	Blood	1
	Male	Not determined	Blood	1
	Female	<6 months	Spleen	1
	Female	6 months–1 year	Spleen	13
	Female	1–2 years	Spleen	10
	Female	>2 years	Spleen	16
	Female	>2 years	Blood	1

**Table 2:** Primers used in this study for the detection of PCMV and PBoV DNA. All oligonucleotides were synthesized by Integrated DNA Technologies (IDT, Coralville, Iowa, USA).

Name	Primer Sequence	Genome Region	Annealing Temperature	Reference
PCMV-F	5′-GCTGCCGTGTCTCCCTCTAG-3′	DNA polymerase	60 °C	[18]
PCMV-R	5′-ATTGTTGATAAAGTCACTCGTCTGC-3′	DNA polymerase	60 °C	[18]
PCMV-P	5′-FAM-CCATCACCAGCATAGGGCGGGAC-TAMRA-3′	DNA polymerase	60 °C	[18]
PBoV-F	5′-TCGAGCTATACAACCGAAGAAGAGA-3′	NP1	60 °C	[14]
PBoV-R	5′-TGTTTCGGAGATGTCCTTGCT-3′	NP1	60 °C	[14]
PBoV-P	5′-FAM-CAGCTCTTCGAATCGCCGCTCTCC-TAMRA-3′	NP1	60 °C	[14]

**Table 3:** Distribution of PCMV-positive wild boar samples across Croatian counties: hunting grounds, demographics, and sample types.

County	Hunting Ground	Gender	Age Group	Sample Type
Koprivnica–Križevci	VI/6 Peski	Female	1–2 years	Blood
	VI/4 Mesarica Plavo	Female	1–2 years	Blood
	VI/9 Repaš	Male	1–2 years	Blood
	VI/107 Prigorje	Male	Not determined	Blood
	VI/1 Dugačko Brdo	Male	6 months–1 year	Blood
	VI/122 Virje	Male	1–2 years	Blood
Grad Zagreb	XXII/25 Grad Zagreb	Female	>2 years	Spleen
	XXII/25 Grad Zagreb	Male	1–2 years	Spleen
	XXII/25 Grad Zagreb	Female	6 months–1 year	Spleen
	XXII/8 Medvednica	Female	6 months–1 year	Spleen
	XXII/8 Medvednica	Male	6 months–1 year	Blood
	XXI/106 Sesvetski Kraljevec	Female	<6 months	Blood
Krapina–Zagorje	II/111 Krapina	Female	6 months–1 year	Spleen
Sisak–Moslavina	III/38 Majdan II	Male	>2 years	Spleen
	III/123 Hrvatska Kostajnica	Female	>2 years	Spleen
	III/124 Divuša	Female	>2 years	Spleen

**Table 4:** Distribution of PBoV-positive wild boar samples across Croatian counties: hunting grounds, demographics, and sample types.

County	Hunting Ground	Gender	Age Group	Sample Type
Krapina–Zagorje	II/127 Oroslavje	Female	6 months–1 year	Spleen
	II/127 Oroslavje	Male	<6 months	Spleen
Sisak–Moslavina	III/40 Zrinska Gora	Male	<6 months	Spleen
	III/20 Majdan I	Male	1–2 years	Spleen
	III/36 Višnjački Bok	Female	1–2 years	Spleen
	III/124 Divuša	Male	6 months–1 year	Spleen
	III/38 Majdan II	Male	>2 years	Spleen

## Data Availability

The original contributions presented in this study are included in the article. Further inquiries can be directed to the corresponding authors.

## Abbreviations

The following abbreviations are used in this manuscript:

ASF	African Swine Fever
PBoV	Porcine Bocavirus
PCMV	Porcine Cytomegalovirus
IPC	Internal Positive Control
RSPS	Reference Weak Positive Serum
Ct	Cycle Threshold
PAC	Positive Amplification Control
NTPC	Non-Target Positive Control
PRV	Pseudorabies Virus
PCV	Porcine Circovirus
PCV2	Porcine Circovirus Type 2
PRRSV	Porcine Reproductive and Respiratory Virus
HEV	Hepatitis E Virus
